# A new service model in East Lothian community learning disability team: evaluation of service with and without specialist positive behaviour support team

**DOI:** 10.1192/bjo.2021.817

**Published:** 2021-06-18

**Authors:** Saima Asif, Andrew McKechanie

**Affiliations:** 1 ST5, NHS Lothian Learning Disability Service; 2 Consultant Psychiatrist, NHS Lothian Learning Disability Service

## Abstract

**Aims:**

To evaluate the provision of services to patients with challenging behaviour in East Lothian Community Learning Disability population with and without specialist behaviour support team.

**Background:**

Behaviour that proves to be a challenge to manage (Challenging behaviour) is not uncommon in adults with intellectual disability and has a reported prevalence of 10–15%.1,2

Positive behaviour support (PBS) is recommended as evidence-based intervention for adults with intellectual disability who have challenging behaviour. East Lothian community learning disability team (CLDT) underwent a change in service model for people with challenging behaviour. This change followed a Health and Social care partnership agreement that behaviour support and management could be provided by multidisciplinary CLDT rather than region-wide specialist team.

**Method:**

Data collection was split into two cycles. First cycle looked retrospectively at six months prior to exit of Specialist Positive Behaviour Support Team (SPBST). Second cycle looked prospectively at 6 months after exit of SPBST.

In first cycle, data were collected doing retrospective review of cases known and referred to SPBST. This included calculating time spent on each individual case by SPBST and by CLDT. SPBST provided information in the form of hours spent on each individual case for patients identified by them. For CLDT, electronic medical records system (TRAK) was used by looking at appointment entries on TRAK. For second cycle, newly developed Complex Behaviour pathway was used to identify the patients. Data were collected by using TRAK system as in the first cycle for CLDT.

Data collected in both cycles was compared at the end of second cycle.

**Result:**

In first cycle, 5 patients were managed jointly by SPBST and CLDT in 96.4 hours over six months and average clinical time spent on each patient was 19 hours. SPBST spent a total of 59 hours and CLDT spent 40 clinical hours. In second cycle, 12 patients were managed by CLDT alone in 130 hours over six months and average clinical time spent on each patient was nearly 11 hours.

**Conclusion:**

Results of this evaluation suggest that SPBST had been providing significant contribution to East Lothian CLDT not only with their expertise but also with clinical time. More than 50 % of total clinical time spent on the patients with challenging behaviour in first cycle, was provided by SPBST. This is also evidenced in second cycle where there is an increase in clinical time of some professions when SPBST was withdrawn.

